# From Mice to Humans

**DOI:** 10.1007/s11892-012-0323-2

**Published:** 2012-09-21

**Authors:** Fiona McMurray, Lee Moir, Roger D. Cox

**Affiliations:** MRC Harwell, Mammalian Genetics Unit, Medical Research Council, Harwell Science and Innovation Campus, Oxfordshire, OX11 0RD UK

**Keywords:** Mouse genetics, GWAS, Type 2 diabetes, Mouse models, FTO, TCF7L2, SLC30A8, CDKAL1, IMPC, IKMC

## Abstract

The genomes of many species have now been completely sequenced including human and mouse. Great progress has been made in understanding the complex genetics that underlie diabetes and obesity in human populations. One of the current challenges is the functional identification and characterization of the genes within loci that are being mapped. There are many approaches to this problem and this review outlines the valuable role that the mouse can play. We outline the mouse resources that are available to the research community, including knockouts with conditional potential for every gene, and the efforts of the International Mouse Phenotyping Consortium to attach phenotype information to these genes. We also briefly consider the potential of TALEN technology to tailor-make new mouse models of specific mutations discovered in humans. Finally, we consider the recent progress in characterizing the GWAS genes *FTO, TCF7L2, CDKAL1,* and *SLC30A8* in engineered mouse models.

## Introduction

Type 2 diabetes clearly has a genetic component and disease susceptibility reflects an interaction between multiple genes, environmental challenges, and age. The strongest challenge is obesity and, for example, European males with a Body Mass Index (BMI) >35 kg/m^2^ and a waist circumference of >102 cm are 22 times more likely to develop diabetes than males with low BMI [1]. Over the past 5 years there has been great progress in identifying human loci associated with diabetes and obesity and many other diseases and traits. Human Genome wide association studies (GWAS) have so far associated over 50 genes with type 2 diabetes (reviewed in [2]). These studies identify single nucleotide polymorphisms (SNPs) that are associated with specific diseases. The identified SNPs may not be directly responsible for the disease but may be in linkage disequilibrium with functional variants that affect a causative gene, likely within the region. Identification of the underlying causative gene is therefore necessary and there are a variety of approaches that may achieve this aim, including the use of mouse models. Often these genes were not previously associated with a disease and in some cases may be of unknown function. Identification of the underlying gene and its function and the pathway and mechanisms it affects, offers the hope of finding targets for therapeutic intervention, regardless of the size of effect of the original mapped locus. A combination of association and expression studies applied to the *KLF14* gene locus in humans also illustrates the potential for discovering novel networks from single starting genes [3••].

This review summarizes new mouse resources and emerging technologies. Further, we highlight recent progress in the utilization of mouse models for the study of human type 2 diabetes loci and which illustrate the potential of the mouse to human (and back again) approach.

## Mouse Models for Diabetes Genes

Diabetes is a complex disease involving many organ systems and multiple signaling pathways; therefore it is necessary to investigate its causes and effects *in vivo*. Mice have been shown to be a strong model for many human diseases including diabetes. Accepting the obvious differences of size and shape and some particular metabolic differences (such as high HDL-C) the mouse is very similar physiologically, anatomically, and genetically to humans. Mice also breed quickly, have a short accelerated lifespan, can be kept in defined conditions, and maintained relatively cheaply.

Standardized protocols for mouse phenotyping are available to help laboratories characterize their models in a comparable way (EMPReSS http://empress.har.mrc.ac.uk/, IMPReSS http://www.mousephenotype.org/impress/, EUMORPHIA http://www.eumorphia.org/). Precise diagnostic criteria that are used to identify the disease in man are not used in the mouse; instead wild-type and mutant mice (age- and sex-matched individuals, which are ideally littermates) are compared with one another. Similar diagnostic tests as those used in human patients have been established in the mouse [4]. This allows phenotyping results to be at least partly comparative between mouse models and human leading to stronger translatable results.

The use of random mutagenesis and exploitation of spontaneous mutations to develop models has been used for many years [5]. However, over the past 20 years or so extensive tools to manipulate the mouse genome have been developed allowing specific mutations to be introduced [6].

## New Mouse Resources

There are a number of resources available to the research community to provide mice for their studies. The Federation of International Mouse Resources (FIMRe) is a collaborating group of mouse repository and resource centers worldwide (Table 1) that archive and provide strains of mice as cryopreserved embryos and gametes, ES cell lines, and live breeding stock (http://www.fimre.org/).

**Table 1:** Members of the Federation of International Mouse Resources (FIMRe)

Member	Link
The Jackson Laboratory (TJL)	http://www.jax.org/
Mouse Mutant Resource Regional Centres (MMRRC)	http://www.mmrrc.org/
Mouse Models of Human Cancer Consortium (MMHCC)	http://emice.nci.nih.gov/
Canadian Mouse Consortium (CMC)	http://www.mousecanada.ca/
Canadian Mouse Mutant Repository (CMMR)	http://www.cmmr.ca/
European Mouse Mutant Archive (EMMA)	http://www.emmanet.org/
RIKEN BioResource Center (RBRC)	http://www.brc.riken.jp/lab/animal/en/
Center for Animal Resources and Development (CARD)	http://card.medic.kumamoto-u.ac.jp/
Australian Phenomics Network (APN)	http://www.australianphenomics.org.a/

Following on from the completion of the mouse genome sequence, that in principal identified every gene in the genome, the International Knock-out Mouse Consortium (IKMC) was developed to systematically generate null mutant embryonic stem (ES) cells that provide “knockout first” alleles that can then be converted into conditional alleles (alleles that are null only from specific time points and/or specific tissues) for every protein coding gene (20,000 plus genes) in the mouse genome ([7•] Table 2). These resources are readily available to the research community [8]. Exploiting the knockout mouse resource the International Mouse Phenotyping Consortium (IMPC, http://www.mousephenotype.org/) has been established and funded to create mouse lines from each targeted ES cell and to determine the phenotype of the resulting mutant mice [9, 10]. Through high-throughput phenotyping of each line, including glucose and body composition traits, using pipelines already established by pilot programs such as EUMODIC (http://www.eumodic.eu/), functional information can be attached to every gene in the mouse genome [10]. This should provide novel genes and models for translational research for human diseases including type 2 diabetes.

**Table 2:** Members of the International Knockout Mouse Consortium (IKMC)

Member	Role
International Knockout Mouse Consortium (IKMC)	Enables the IKMC database to be searched for a gene of interest by gene symbols, gene IDs or genome location. Provides information on availability of knockout attempts and other resources. Incorporates information from all the partners and provides a “one stop shop” for information on knockouts for your gene of interest.
http://www.knockoutmouse.org/	
NIH Knockout Mouse Project (KOMP)	Incorporates KOMP-CSD and KOMP-Regeneron, Inc.
http://www.nih.gov/science/models/mouse/knockout/	KOMP-CSD use promoterless and promoter-driven targeting cassettes to make a 'Knockout-first allele' and produce frame shift mutations of a ‘critical’ exon.
	KOMP-Regeneron Velocogene constructs generate complete null alleles that delete the entire protein coding sequence of the target gene.
	Provides central database resource through the Knockout Mouse Project Data Coordination Centre (KOMP DCC).
	KOMP Repository to archive, maintain and distribute KOMP products.
The European Conditional Mouse Mutagenesis Program (EUCOMM)	Uses conditional gene targeting and trapping approaches to generate 12,000 conditional mutants as ES cells.
www.eucomm.org/	
North American Conditional Mouse Mutagenesis project (NorCOMM)	Utilizes a combination of gene trap random mutagenesis and systematic high-throughput targeting of remaining un-trapped genes.
http://www.norcomm.org/index.htm	
Texas A&M Institute for Genomic Medicine (TIGM)	A huge resource of over 350,000 gene trap C57BL/6N
http://www.tigm.org/	ES cell lines for more than 13,000 mouse genes.
	In addition holds 270,000 sequence-tagged 129/SvEvBrd gene trap embryonic stem cell clones.

## Mouse Models for Specific Human Mutations–New Technology

Transcription activator like effector nucleases (TALENs) were first reported in 2010 [11]. They are very similar to Zinc finger nucleases (ZFNs), binding to a particular DNA sequence and then causing a double stranded DNA break, the cell then utilizing homologous recombination that allows a homologous donor sequence to be introduced, and used as a template for DNA repair thus integrating a desired sequence (carrying a tailored modification) into the genome, or in the absence of a donor sequence error prone non-homologous end-joining that may lead to deletions or insertions that may result in a null allele [12–14]. These manipulations can be carried out in zygotes and substantially speeds up the process of making a tailored mutant mouse. This is a very efficient process but is limited by the repertoire of ZFNs and the potential for off target mutations. However, TALEN technology may be more flexible and have reduced off target events. TALENs are made of DNA binding modules that each recognizes a single nucleotide and thus it is possible to assemble a protein that recognizes a sequence of choice (reviewed [15]). This protein can then be coupled with a nuclease or another modifying protein for specific delivery to a unique site in the genome (reviewed [16]). In the last year TALENs have successfully been used to engineer site specific mutations in yeast, zebra fish, rats, plants, and human pluripotent stem cells [17]. The ability to introduce precisely targeted changes at high efficiency has many applications, including introducing human mutations into mouse genes quickly and precisely, and may also find utility in the manipulation of single exon genes where there is a risk of disturbing important flanking sequences by conventional targeting methods, especially if a conditional allele is required. These technologies hold the promise of targeted gene correction in human induced pluripotent stem cells, as has been recently demonstrated for correcting a biallelic point mutation in α-1-antitrypsin, which in man causes a deficiency which leads to emphysema [18].

## Genes in Man and Mouse

**Fig. 1:**
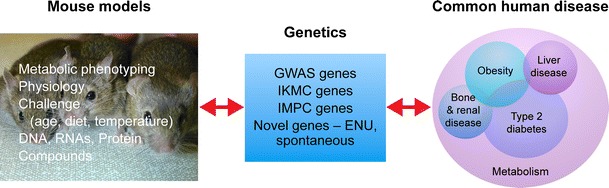
From mice to humans. Mouse models allow specific genes to be manipulated and tested for their effects on metabolism and physiology. These models can also be challenged in a number of ways to reveal phenotypes, for example by feeding a high fat diet. These models can provide tissues for physiological analysis and RNA, DNA, and protein for molecular analysis of any tissue at any stage of development or challenge. Suitable models can also be used for testing compounds that may target specific genes and pathways, in the search for therapeutic opportunities. The underling genes for testing may come from GWAS studies; they may also be identified in mouse mutagenesis programs as giving rise to relevant disease traits. Patients carrying mutations and polymorphisms in genes inform the genetics and allow cross correlation of the metabolic and physiological effects in models, directing the use of models and testing their validity

To illustrate the application of mouse genetics in going “from mice to humans” we will now consider 4 genes from human diabetes GWAS examined in recent studies; *FTO, TCF7L2, CDKAL1,* and *SLC30A8* (Fig. 1), all of which had not previously been considered as prominent candidates for diabetes research until uncovered by GWAS.

### FTO

In 2007 a T2D GWAS identified a strong signal at rs9939609, in intron 1 of the *FTO* gene. This association was due to an increased body mass index (BMI), and although the SNP was intronic, the gene where it was found was of unknown function, and was a close neighbor to *RPGRIP1L* (formerly *FTM*) [19, 20]. Individuals homozygous for the risk allele weigh on average 3 kg more than those individuals without it, and since the identification of this SNP several groups have worked on this region to understand the cause of the increased BMI, and several different mouse models have been generated.

A preexisting model with a large 1.6 Mb deletion of 6 genes including *Fto* had severe developmental defects with homozygote mice dying during gestation [21]. This model although interesting was not specific enough to understand the function of *Fto*. A knockout mouse was generated by Fischer and colleagues. Homozygous knockout mice are lean, with a mild insulin sensitivity improvement, but they are also growth retarded [22]. Another knockout from Gao et al. had a similar phenotype, and this group also generated a nervous system specific knockout using a general neuronal Cre recombinase (driven by the nestin gene promoter) to delete a floxed exon 3 in nestin expressing cells and obtained a similar phenotype [23]. They concluded that *Fto* functions in the central nervous system to regulate postnatal growth [23]. Screening the ENU DNA archive (male mice carrying ENU induced point mutations stored as tissues, DNA, and frozen sperm for IVF) at MRC Harwell led to the identification of a dominant point mutation in *Fto* (FTOI367F) in exon 6 of the gene. Mice homozygous for this mutation are lean and do not show perinatal lethality or an effect on body length [24]. Finally Church and colleagues also generated a mouse which conditionally overexpresses the *Fto* gene from the Rosa26 locus using the CAGGs promoter (chicken b-actin promoter) [25]. Mice carrying extra copies of the gene have a dose dependent increase in body weight due to increased adipose tissue mass and adipocyte size. These overexpression mice are hyperphagic and when fed a high fat diet have an increased glucose tolerance and increased fasting insulin.

These mouse models suggest that *FTO* plays a role in control of body weight and composition. Therefore the identified SNP could affect FTO function or expression. Although *FTO* is not a direct candidate for T2D, it does increase the risk for becoming overweight. This can be seen in the overexpression mouse model, as when these animals are fed a high fat diet they have a decreased glucose tolerance. These mice will therefore be a useful model for obesity and T2D translational studies.

*FTO* is an excellent example of how by using mouse models it has been possible to validate a GWAS identified gene of unknown function associated to obesity and T2D. This approach can therefore be applied to other GWAS loci, to identify the underlying genes [26].

### *TCF7L2*

The *TCF7L2* locus was initially mapped as a suggestive locus with linkage to chromosome 10 in an Icelandic population and then confirmed in a second study [27, 28]. Subsequent GWAS have shown *TCF7L2* to be a consistent strong signal in many studies and exhibits one of the largest effects (odds ratio per allele about 1.37) to date (see in particular [20, 29, 30], and reviewed [31]). Data from individuals carrying risk alleles indicated lowered insulin secretion [32]. Work with isolated islets and insulin secreting cell lines has shown that loss of *TCF7L2* leads to decreased insulin secretion in response to glucose and incretins [33–35].

What have mouse models contributed to the understanding of this gene that had already been validated *in vitro*? A constitutive knockout of this gene leads to lethality shortly after birth due to intestinal defects and thus precludes an analysis of glucose homeostasis in these animals [36]. Savic et al. (2011) have carried out 3 types of experiment using mice [37••]. In one they used human bacterial artificial chromosomes (BACs, large segments of DNA usually in the 100–150 kb range) engineered to express blue LacZ to test enhancer driven expression in embryos (visualizing the blue staining) and demonstrated that the genomic interval encompassing the diabetes associated SNPs drive expression in relevant tissue such as intestine and pancreas (although surprisingly not in adult islets), in contrast to BACs outside of the region. Further an engineered deletion of the associated region from the expressing BAC abolished expression, thus pointing to the presence of control sequences within this region, and affecting the expression of *Tcf7l2* (see also a follow up study by Savic et al. [37••, 38]). In the second experiment they made a *Tcf7l2* knockout using zinc finger nucleases to cause a frameshift in exon 11. As observed for the constitutive knockout, homozygous null mice died soon after birth. However heterozygous null mice showed improved glucose tolerance and reduced insulin on a standard and high fat diet. Finally, Savic et al. (2011) took a human BAC with a mouse cDNA recombineered into the TCF7L2 translation start site and generated a series of mice overexpressing *Tcf7l2*. These mice exhibited glucose intolerance on a high fat diet. Thus these experiments demonstrate that the associated SNPs are in regulatory elements (although perhaps not for adult islets at least in the context of a randomly integrated transgenic BAC) and that manipulating *Tcf7l2* expression modulates glucose tolerance, low expression improving and higher expression impairing glucose tolerance. Another group using a knockout first allele from the IKMC confirmed the perinatal homozygous lethality of null mice and improved glucose tolerance in heterozygous mice [39]. Significantly, Gaulton et al. (2009) have shown that the chromatin of the TCF7L2 intronic variant is in an islet-specific open conformation [40]. Further, that the diabetes associated rs7903146 T allele chromatin is in a more open conformation than the C allele. An open conformation indicates active gene regulatory elements that may affect transcriptional rates, promoter usage, and differential splicing. Reporter assays demonstrated increased enhancer activity of the T allele compared with the C allele in beta cell lines [40]. These data are consistent with increased expression from the risk allele in islets leading to increased diabetes risk and is consistent with the above mouse studies.

In the studies described above all of the manipulations are present in the germline and then all tissues. In order to address the question whether selective deletion of *Tcf7l2* in pancreas impairs or improves glucose homeostasis and insulin secretion Xavier et al. carried out a conditional knockout using *Pdx1* promoter Cre recombinase and a floxed exon one [41••]. Mice carrying both alleles specifically and homozygously deleted exon 1 during early endocrine pancreas development and were compared with mice carrying the Cre only. When challenged with an intraperitoneal glucose bolus these mice showed age dependent glucose intolerance by 20 weeks of age. In an oral glucose tolerance test, impairment was detectable from 12 weeks of age; in this test the incretin response (peptides secreted from the gut that amplify insulin secretion by the beta cell) contributes to glucose tolerance. Isolated islets showed impaired glucose stimulated insulin secretion and GLP-1 (an incretin) response. These pancreas specific knockout phenotypes are consistent with the *in vitro* human and mouse islet and cell line siRNA-mediated silencing experiments. However, they contrast the heterozygous global constitutive mouse knockouts and overexpression experiments and the active chromatin experiments in human islets that were described earlier. It is clear that *Tcf7l2* is expressed in multiple tissues and therefore in the other models glucose tolerance may be affected by changes in other tissues as those manipulations were not tissue specific, unlike the Xavier et al. experiment where there is deletion in islets alone. Although *Pdx1* driven Cre will delete in other islet endocrine cells, no difference was observed in circulating glucagon or GLP-1 levels strongly implicating beta cells. Another possible explanation for the discrepancies is that there is a high degree of tissue specific alternative splicing of the *TCF7L2* gene [42–45]. In these various experiments the *Tcf7l2* gene is manipulated in different ways and each may alter the tissue specific splicing of the gene and in the case of the cDNA overexpression experiment only 1 isoform is represented. It will be interesting to evaluate the different mRNA isoforms expressed in each of these situations.

The mouse *in vivo* experiments clearly confirm the importance of *Tcf7l2* in glucose homeostasis but reveal a complex picture where more than 1 tissue may be relevant. The complete loss of the gene in islet cells definitively impairs insulin secretion and incretin response. Further tissue specific manipulation of expression will be very interesting and will contribute to our understanding of gene and tissue specific function.

### *CDKAL1*

CDK5 regulatory subunit associated protein 1-like 1 (CDKAL1) is homologous to CDK5RAP1, an inhibitor of cyclin-dependent kinase CDK5, which can transduce glucotoxicity signals in pancreatic β-cells. *CDKAL1* has been recently associated to T2D by several GWASs [20, 29, 46–48] and replicated in many other studies, it has also been associated with low birth weight in those carrying the risk allele [49].

Despite the initial, homology observation the biochemical function of CDKAL1 was unclear, however, further bioinformatic sequence analysis, and functional assays identified CDKAL1 as the first eukaryotic member of the methylthiotransferase family (MTTase) to be found so far [50]. Using a mouse knockout Wei et al. were able to demonstrate that *Cdkal1* specifically modifies the tRNA for lysine from t^6^A (N6-threonyl carbamoyl adenosine) at position 37 to ms^2^t*6*A (2-methylthio-N6-threonyl carbamoyl adenosine), a modification specific to this tRNA and essential to prevent codon misreading [51••]. *Cdkal1* gene-trap knockout mice have impaired β-cell function, showing impaired glucose stimulated first-phase insulin release *in vitro* [52]. Wei et al. [51••] used a β-cell specific knockout of *Cdkal1,* finding islet hypertrophy, decreased insulin secretion, and impaired blood glucose control. They also observed that misreading of a Lys codon in proinsulin occurred, resulting in a reduction of glucose stimulated proinsulin synthesis. This reduced translation accuracy, causes the synthesis of abnormal insulin, which triggered ER stress in β-cells. The adverse effects of this mutation were exacerbated by high fat diet feeding. These studies demonstrate a clear link between this gene within a GWAS locus and insulin secretory defects that may at least partly explain the association with type 2 diabetes.

### *SLC30A8 (ZnT-8)*

Solute carrier family 30, member 8 (SLC30A8) encodes a ZnT8 zinc transporter, expressed highly in the pancreas, and regulates zinc efflux [53]. It has been associated with fasting glucose levels and T2D risk in several GWAS studies [30, 47, 54]. Recently Kanoni and colleagues conducted a large-scale gene-diet interaction meta-analysis. They identified a nominally significant interaction between total zinc intake and the *SLC30A8* rs11558471 variant on fasting glucose levels. This suggests an inverse association between total zinc intake and fasting glucose in individuals carrying the glucose-raising A allele [55].

Several groups have generated global knockout ZnT8 mice, which have been a useful tool for investigating its link with T2D. Two groups have looked in parallel at an exon 1 knockout mouse line maintained as separate colonies and both found effects on zinc accumulation by β-cells, glucose intolerance and insulin secretion that were diet, age, and genetic background dependent [56–58]. Interestingly insulin secretion in isolated islets was either preserved or enhanced [56–58]. This model is useful as it may parallel disease in humans where for example, obesity may result in increased demand on β-cells for insulin that leads to a chain of events resulting in impairment of insulin secretion, and because of predisposing gene variants, like *ZnT8*, diabetes [56–58]. Another group has made an exon 3 knockout finding similar effects on zinc metabolism and normal glucose tolerance; it would be interesting to examine the effect of high fat diets on these mice [56]. In contrast to the exon 1 knockouts islets from these mice showed impaired glucose stimulated insulin secretion that may again reflect age, diet, and genetic background effects [56]. The *ZnT8* gene is expressed in α-cells (secreting the counter-regulatory hormone glucagon) and a number of other tissues in addition to β-cells. Conditional knockout mice have been used to specifically knockout the gene in α or β-cells alone to address the contribution of each cell type. Again using the exon 1 floxed allele, mice were crossed with rat insulin promoter Cre recombinase line (to delete exon 1 in β-cells), or a glucagon promoter Cre (to delete in α cells), and the result was that the β-cell knockout mice showed many of the original features of the global knockout including glucose intolerance and a reduction in glucose stimulated insulin secretion, whilst in contrast, α cell deletion showed no abnormalities in plasma glucagon levels or glucose homeostasis [59•]. These experiments demonstrate that loss of function of this gene in the β-cell, rather than in α cells, gives rise to the diabetes association of this gene. In another study a β-cell knockout was compared with whole body knockout mice that had been fed a high fat diet. The β-cell knockout mice developed glucose intolerance as described before, but remained similar in weight to control mice. In contrast, whole body deletion leads to obesity and severe insulin resistance [60]. These experiments suggest both β-cell specific and non-β-cell tissue specific effects involving the ZnT8 gene and type 2 diabetes risk [60].

## Conclusions

The mouse has proven to be a useful model system for the exploration of the genetics of diabetes and has a role to play in translating the results of GWAS (loci that contain genes that are relevant to human disease susceptibility), alongside other approaches. As the specific examples described above demonstrate, these experiments can provide valuable insights that are relevant to human studies. This technological approach can be used to identify underlying culprit genes within loci where this is unknown and also to eliminate candidate genes and so functionally “fine map” an association signal. As with any model system there are going to be differences when comparing to heterogeneous human populations and that is why mouse genetics needs to be part of an integrated approach that includes human patient studies where possible. Projects such as IKMC and IMPC provide a resource of knockout mice that means these approaches are accessible to any group with access to a mouse facility. The phenotyping of knockout genes and the availability of that data in public databases will undoubtedly reveal new targets in the field of diabetes that can be tested for a role in patients. New technologies are adding to our ability to manipulate genes in the mouse and indeed to contemplate human gene therapies.
